# Tracheal replacement by autogenous aorta

**DOI:** 10.1186/1749-8090-4-23

**Published:** 2009-06-09

**Authors:** Farhad Anoosh, Hossain Hodjati, Seifollah Dehghani, Nader Tanideh, Perikala V Kumar

**Affiliations:** 1 Department of Surgery, New York Hospital Queens (Weill College of Cornell University), New York, USA; 2 Department of Surgery, Shiraz University of Medical Science, Shiraz, Iran; 3 Department of Surgery, School of Veterinary Medicine, Shiraz University, Shiraz, Iran; 4 Department of Pharmacology, Shiraz University of Medical Science, Shiraz, Iran; 5 Department of Pathology, Shiraz University of Medical Science, Shiraz, Iran

## Abstract

**Background:**

Tracheal defects may occur after trauma or prolonged intubation. Resection of tracheal tumors also poses a major challenge for substitution. In an effort to solve this problem, different techniques have been tried with little success. We report on a new animal model which showed acceptable results with fewer complications.

**Methods:**

We replaced 5 cm of cervical trachea in 10 dogs with harvested infra-renal aorta and repaired the aortic defect with Dacron graft.

**Results:**

Necropsy of the grafted aorta and anastomotic site revealed well healed anastomosis in all animals together with ciliated columnar epithelium coverage of grafted aorta and neovascularization of aortic wall.

**Conclusion:**

Aortic graft is preferable to other substitutes because of less antigenicity, less vascularity, and no mucous secretions or peristalsis

## Introduction

Tracheal defects may occur after trauma or prolonged intubation. Resection of tracheal tumors also poses a major challenge for substitution. In an effort to solve this problem, different techniques have been tried with little success. The various tracheal substitutes and techniques of reconstruction were analyzed by Grillo [1], who classified them in five categories: foreign materials, nonviable tissues, autogenous tissues, tissue engineering, and tracheal transplantation. Attempts with foreign materials led to problems of chronic infection, airway obstruction, migration of the prosthesis, erosion of major blood vessels and proliferation of granulation tissue. Implantation of nonviable tissues, either chemically treated, frozen or lyophilized has been associated with poorly functional results. Reconstructions with autogenous tissues such as skin, fascia lata, pericardium, costal cartilage, bladder, esophagus or bowel are complex procedures, which have been associated with disappointing results. More recently, efforts have been made to induce the formation of cartilaginous tubes covered with epithelial cells, but to date this type of tissue engineering has not provided reliable results. Finally, tracheal allotransplantation has been also disappointing so far due to complication of necrosis or stenosis of the graft. In addition immunosuppressive therapy does not permit a clinical application in the treatment of cancer. As Grillo suggested: "We must continue to maintain an open mind about this intriguing but thus far unsolved surgical dilemma – replacement of the tracheal conduit" [2]. Recently, Martinod and colleges have used autogenic aortic graft [3,4] and allogenic Aortic grafts [5] to replace long segments of tracheal defect and Carina [6] using sheep as animal model with promising results. Seguin and colleges have used cryopreserved, decellularized aortic allograft supported by temporary stent to prevent airway collapse [7]. We report on a new animal model, which showed acceptable results with few complications.

## Methods

Eleven healthy dogs ranging from 18 kg to 24 kg in weight were studied. All animals received care in compliance with the "Guide for the care and use of laboratory animals" prepared by the Institute of laboratory animal resources, National research Council, and published by the National Academy Press, revised 1996. Five centimeter of cervical trachea was resected and replaced by harvested abdominal aorta. The aortic defect was repaired with Dacron graft.

### Anesthesia

General anesthesia was induced with thiopental and maintained with oxygen and 1–3% halothane through an endotracheal tube. All animals were perfused with a crystalloid solution. Pulse were monitored Introperatively. One gram of Cephazoline was injected after the induction of general anesthesia for wound prophylaxis.

### Surgical Procedure

**Figure 1:**
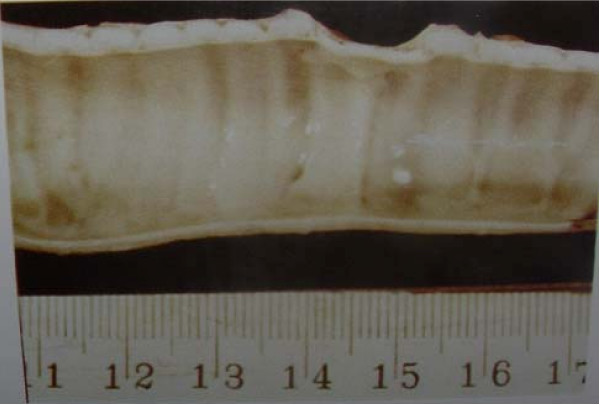
**window-shape healed aortic graft on the trachea**.

A laparatomy was performed thorough a midline abdominal incision to expose at least 5 cm of infrarenal abdominal aorta. Before aortic clamping, 100-unit/kg heparin was given intravenously and 5 cm of aorta was resected circumferentially and replaced by Dacron graft using a running 4-0 Polypropylene suture. The average time for the harvesting and anastomosis was less than 20 minutes without a need for aortic shunt. The excised aortic segment was placed in heparinized blood solution. The abdomen was then closed in two layers by 0-0, 3-0 nylon sutures. Access to the cervical trachea was gained through a longitudinal vertical neck incision by splitting the anterior neck muscles. The trachea was exposed and a 5 cm long window shape defect is made with resection of anterior tracheal rings. The posterior membranous portion of trachea left intact. A longitudinal incision was made along the whole length of harvested aorta [Fig. 1] and while the endotracheal tube left in place, the aortic graft placed over the window shaped defect of cervical trachea by continuous suturing with 4-0 Polypropylene. The aortic graft maintained its consistency and there was no need to place a stent in the lumen of aortic graft. The cervical wound was closed in layers with 2-0 chromic and nylon 3-0 (m). The animals were extubated after emerging from anesthesia and placed under close observation.

### Clinical evaluation

In post operative day one diet started for the animals and advanced. Clinical examination was performed daily for fist two week post op and then every month including overall status, weight, respiratory status and injury to the laryngeal nerve. Because none of the animals developed respiratory problems or fever and there was not stent, bronchoscopy and chest X-ray was not performed in the post operative period.

### Histologic evaluation

Animals were sacrificed between two and six months after the procedure with intravenous injection of potassium chloride and Propofol to remove the grafts. En block resection of the trachea and the graft with surrounding tissues was performed and sent for macroscopic and microscopic examinations. Post mortem specimens were immediately placed in 10% formaldehyde solution for preservation. The specimens were evaluated for patency, microscopic evidence of inflammation and morphologic transformation of the graft.

## Results

Ten animals survived the procedure. The average operative time was 105 minutes. Between 4 to 5 tracheal rings removed in each case. One dog died 4 hours after the operation secondary to heart failure. All the animals started on diet the first post operative day. All abdominal wounds healed primarily. There was one cervical wound infection, which was healed with secondary intention. There was no clinical respiratory problem, cough or pneumonia. There was no evidence of hoarseness and all of the animals start barking a few days after the operation. There was no complication related to aortic Dacron graft. None of the animals developed claudication.

**Figure 2:**
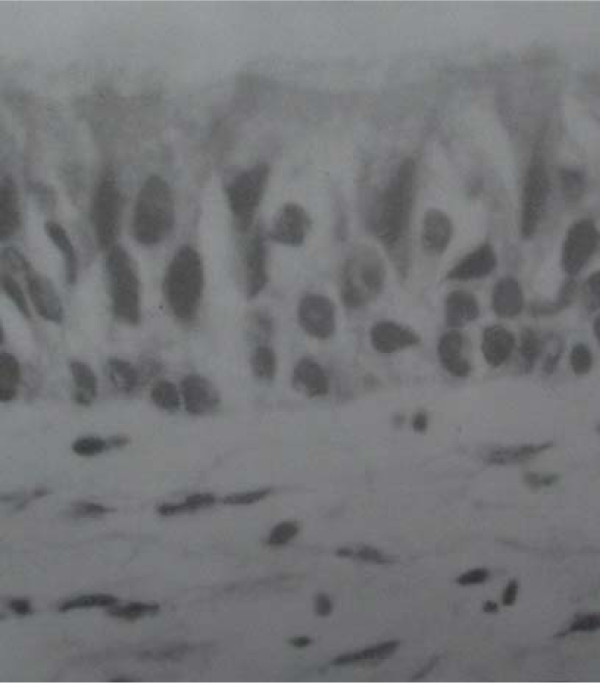
**Ciliated Columnar epithelium over the grafted aorta**.

**Figure 3:**
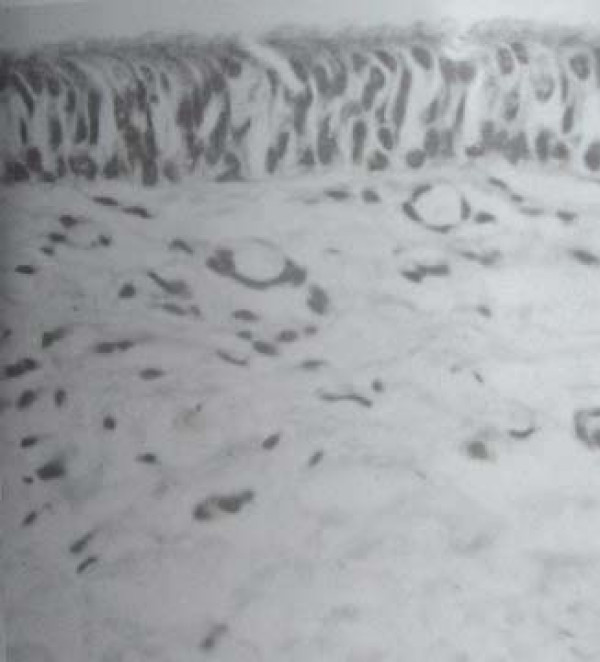
**Neovascularization of grafted aortic wall**.

At necropsy, gross examination showed a patent lumen in all specimens. The external surface was smooth and had area of depression at the site of graft. There was no abscess formation or sing of infection in the neck. There was evidence of adhesion of adjacent muscles to the trachea. There was evidence of neovascularization from the adjacent muscles to the aortic graft. Microscopic examination of H&E stained sections of grafted aorta and the anastomotic site revealed well healed anastomosis in all animals. There were areas of mild chronic inflammation, fibrosis, foreign body granuloma and squamous metaplasia in the animals sacrificed within two months of operation. In the animals sacrificed longer that 2.5 months, Pathology revealed no foreign body granuloma, minimal inflammation and ciliated columnar epithelium coverage of grafted aorta and neovascularization of aortic wall [Fig 2 and Fig 3], [Table 1].

**Table 1:** Follow up, complications, functional status and histological examination of specimens

Animal	Follow-up	complication	Stenosis	Functional status	Histo- pathology
1	1 mo	None	0	Excellent	Squamous interrupted

2	1 mo	Wound infection	20%	Excellent	squamous interrupted

3	2 mo	None	0	Excellent	squamous continuous

4	2 mo	None	10%	Excellent	Squamous interrupted

5	2.5 mo	None	0	Excellent	Mixed squamous/mucociliary

6	2.5 mo	None	20%	Excellent	Squamous continuous

7	3 mo	None	0	Excellent	Mixed squamous/Mucociliary

8	3 mo	None	10%	Excellent	Mixed squamous/Mucociliary

9	6 mo	None	0	Excellent	Continuous mucociliary

10	6 mo	None	20%	Excellent	Continuous Mucociliary

11	1 day	Mortality	N/A	N/A	N/A

## Discussion

Studies on prosthesis for repairing the tracheal defects have a long history beginning with Daniel's experiment in 1948. Since then, a variety of prosthesis has been used, but the results have been disappointing in a significant proportion of cases. In an extensive analysis of the literature, Grillo [1,8] underlined the main technical difficulties and complications associated with the current surgical approaches to tracheal replacement in five categories: foreign materials [9], nonviable tissues [10-12], autogenous tissues [13-16], tissue engineering [17], and tracheal transplantation. Complications associated with prosthetic grafts were mostly related to lack of biocompatibility and included local infection, anastomotic dehiscence, vascular erosion, granulomatous lesions, and stenosis. Complications associated with biologic tissues were related to the ischemic injury of the harvested graft leading to necrosis or stenosis. None of the proposed tracheal substitutes has produced consistent results that would standardize a clinical approach. Vascularized autografts such as pectoralis muscle, esophagus, and jejunum have shown promising experimental results when used alone, but it is unlikely that the soft tissue alone could provide adequate long-term structural support to maintain a patent airway. Composite implants consisting of a revascularized jejunal autografts in combination with a permanent rigid implantation stent have been utilized in a canine model with promising result. Cryopreserved autografts have been performed, first by Tojo et al. on rats and then by Jacops et al. on pediatric allograft. In both instances, it was found that cryopreservation reduced the antigenicity of the transplants. Martinod and associates have used aortic autograft and allograft with temporary stenting in sheep with promising results. They have reported the tissue transformation using aortic graft. Their findings raise the question of how the aortic tissue can transform into an almost normal trachea within 6 months, with the hypothesis that the inflammatory response created favorable conditions for reepithelialization and subsequent cartilage neoformation from stem cells triggered by environmental factors. Their study also shows a progressive transformation of the vascular graft into a structure resembling the tracheal tissue. This transformation was responsible for the long-term patency of the trachea and its normal function up to 3 years.

In our study, the aorta as an autogenous tissue was used to replace the trachea in a dog model. Surgical techniques for aortic segment excision and Dacron replacement with aortotracheal graft are not complicated and feasible to be done in one stage. Secondly, the aorta has no autogenous immunity-related problems and its diameter in human is comparable to the trachea. Furthermore it provides a sufficient diameter for airway patency. Due to disparity between tracheal and the aortic diameter in dogs, the aorta was transplanted to the proximal and distal tracheal and posterior membranous parts, but it appears that no such disparity is anticipated in humans. The gross and microscopic study of transplanted aortic segment showed neovascularization from adjacent tissues providing a good and resistant grafted tissue without vascular pedicle. The growth of endothelial ciliated cells over the grafted aortic segment lumen without mucous secretion and luminal stenosis is another positive point for this method in comparison with other tubular grafts such as esophagus, jejunum and collagen-conjugated mesh

## Conclusion

In conclusion, this study confirms that an autogenous aortic graft is a valuable substitute for tracheal replacement because of less antigenicity, less vascularity, and no mucous secretions or peristalsis. It also shows a progressive transformation of the vascular graft into a structure resembling the tracheal tissue. We did not have any aortic surgery related complications in our study, but it may subject the patient to potential complications of aortic surgery. The use of an arterial allograft may be more practical in human, but it remains to be demonstrated that the same tracheal neogenesis and the same long-term function can be obtained in spite of the allogeneic nature of the graft.

## Competing interests

The authors declare that they have no competing interests.

## Authors' contributions

FA participated in design of the study, carried out the Coordination, collecting and analyzing the data, getting grant for the study, performing the operations and post operative care, writing the manuscript.

HH conceived the study, participated in design of study, supervised the study and operative procedures and reviewed the manuscript.

SD participated in design of the study, anesthesia in the operative room, the operative procedures and post-operative care.

NT participated in coordination for operative procedures and post operative care in animal lab.

PK carried out the gross and microscopic evaluation of pathology.

All authors read and approved the final manuscript.
